# Experimental winter warming increases activity with signs of potential DNA damage in common wall lizards

**DOI:** 10.1242/jeb.251440

**Published:** 2025-11-21

**Authors:** Miary Raselimanana, Wolfgang Wüster, Jonathan D. Blount, Christopher A. Mitchell, Rhys Morgan, John W. Wilkinson, Kirsty J. MacLeod

**Affiliations:** ^1^ School of Environmental and Natural Sciences, Bangor University, Deiniol Road, Bangor, Gwynedd LL57 2UW, UK; ^2^ Centre for Ecology and Conservation, Faculty of Environment, Science & Economy, University of Exeter, Penryn Campus, Penryn, Cornwall TR10 9FE, UK; ^3^ Amphibian and Reptile Conservation, 744 Christchurch Road, Boscombe, Bournemouth, Dorset BH7 6BZ, UK

**Keywords:** Fluctuating temperature, Hibernation, Overwintering behaviour, Oxidative stress, *Podarcis muralis*

## Abstract

Winter is warming faster than summer, posing a substantial threat to hibernating ectotherms, whose physiology depends directly on environmental conditions. While the effects of active season warming are increasingly well understood, the consequences of winter warming remain understudied. Research has predominantly focused on single, constant thermal regimes, overlooking the role of thermal variability. Furthermore, the specific warming patterns most disruptive to dormancy, their effects on winter activity and the subsequent physiological consequences are poorly understood. Here, we experimentally manipulated overwintering temperatures in the common wall lizard (*Podarcis muralis*), from a population introduced in southern UK, to assess the effects of different winter warming regimes on activity, body condition and oxidative stress. Lizards were exposed to three treatments for 3.5 months: a typical cold winter (4±1°C), a constant mild winter (8±1°C) and a fluctuating winter temperature (5 days cold: 4±1°C; 2 days mild: 8±1°C). Constant mild warming significantly increased activity, whereas the fluctuating regime did not, suggesting a temperature-duration threshold for full arousal. Despite increased activity, body condition, total antioxidant capacity and lipid peroxidation remained largely unaffected, indicating limited physiological disruption. However, the mild regime showed a trend toward increased oxidative DNA damage, highlighting a previously unrecognised physiological vulnerability that merits further investigation. Overall, our findings suggest behavioural resilience of common wall lizards to moderate winter warming, though hidden molecular costs could emerge under sustained mild conditions. We encourage integrating behavioural sensitivity and subtle physiological responses into models predicting species resilience to climate change.

## Teaser

**Highlighted Article:** Winter warming makes hibernating wall lizards more active, with signs of DNA damage after emergence, but no change in body condition, revealing subtle costs of milder winters.

## INTRODUCTION

Global warming has significantly increased mean air temperature, threatening ecosystems and shifting countless species toward extinction (Pörtner et al., 2023). While much attention has focused on rising summer temperatures and their impacts for wildlife – such as heat stress, range shifts and altered reproductive timing (Bellard et al., 2012; Dayananda et al., 2021; Pacifici et al., 2015; Walther, 2010) – winter temperatures have increased at a faster rate and exhibit greater variability (Intergovernmental Panel on Climate Change, 2014; Vose et al., 2005). Yet, the effects of milder winters on animals remain underexplored, leading to a seasonal bias that impedes a comprehensive understanding of the impacts of climate change on biodiversity (Moss and MacLeod, 2022; Williams et al., 2015).

Winter warming is likely to be particularly disruptive for terrestrial temperate species (Williams et al., 2015). Many of these species overwinter by entering complete or partial dormancy to conserve energy during periods of low resource availability (Blix, 2016; Wilsterman et al., 2021), a process that is largely regulated by thermal cues. When these cues are disrupted, as in a mild winter, temperatures may become too cold for sustained activity yet insufficiently cold to maintain dormancy. Consequently, mild winters are predicted to have a range of detrimental/disruptive effects on overwintering animals (Reading, 1998; Williams et al., 2015). Documented consequences include reduced cold tolerance in overwintering insects (Mikucki and Lockwood, 2021), increased freeze–thaw related mortality in freeze-tolerant species (Marshall and Sinclair, 2012), elevated metabolic rates and energy use in overwintering bats (Day and Tomasi, 2014) and turtles (Muir et al., 2013), increased midwinter arousal/emergence (Nordberg and Cobb, 2016, 2017), disruption of endocrine pathways modulating reproduction (Lutterschmidt et al., 2022), and shifts in phenology – such as advances in mid-latitude animals (Cohen et al., 2018) or delays in insects (Marshall et al., 2020).

Nevertheless, it remains unclear which specific climatic drivers – such as shifts in mean temperature or increased thermal variability – are most disruptive to overwintering biology and more likely to induce stress in organisms (Williams et al., 2015). Most empirical studies of overwinter temperature effects on reptiles to date have focused on a single driver, particularly constant high-temperature regimes (e.g. Brischoux et al., 2016; Clarke and Zani, 2012; Devlin et al., 2022), with only a few exploring the role of thermal variability (Slein et al., 2023). Even fewer studies have tested these drivers in combination (but see Verheyen and Stoks, 2019), which would allow us to compare their effects and identify which driver is more likely to trigger disruptions. Additionally, much of our understanding of the physiological effects of fluctuating temperatures comes from research conducted during the active season, not winter. For instance, compared with constant temperature regimes, temperature fluctuations have been shown to elevate stress levels and reduce immune function in rattlesnakes (*Crotalus durissus*) (Fabrício-Neto et al., 2019), and to increase stress-related gene expression and shorten lifespan in aquatic ectotherms (Stocker et al., 2024). Whether similar effects occur – or are amplified – under winter conditions remains poorly documented.

Squamate reptiles are widely recognised as among the most climate-sensitive vertebrates: early projections estimated that nearly 40% of species could face extinction by 2080 due to warming (Sinervo et al., 2010), and subsequent global assessments and trait-based analyses also highlight their high sensitivity to climate change (Böhm et al., 2016; Hayden Bofill and Blom, 2024; Mi et al., 2022). This high sensitivity is driven not only by direct temperature rise effects but also by an increasing ‘cost-of-living’ squeeze during the active season, where warming increases energy demands while potentially reducing foraging opportunities (Huey and Kingsolver, 2019; Mizsei et al., 2025; Wild et al., 2025). Similar pressures may also apply during the winter months, when many temperate reptiles undergo a period of temperature-dependent dormancy. During winter dormancy, reptiles rely on energy reserves accumulated in the active season to survive prolonged periods of low food availability (Bonnet et al., 1998; Shine, 2005). Prolonged cold exposure also initiates physiological processes required for successful reproduction in spring (Licht et al., 1969; Lutterschmidt et al., 2022). Winter warming is however likely to disrupt reptile hibernation (Moss and MacLeod, 2022): for example, a short-term increase in winter temperature can interfere with biological rhythms and encourage midwinter emergence, as observed in timber rattlesnakes (*Crotalus horridus*) (Nordberg and Cobb, 2016, 2017). Such premature activity can be costly as elevated metabolic rates increase energy use when food is scarce or unavailable, and even if prey is present, digestion may be impaired by low temperatures (Harlow et al., 1976; Huey and Kingsolver, 1989; Sperry and Weatherhead, 2012). Disruptions to hibernation caused by winter warming can have profound consequences, with increased energy expenditure accelerating depletion of energy reserves and leading to poorer body condition at emergence (Moss and MacLeod, 2022). This effect has been observed in aspic vipers (*Vipera aspis*), where elevated energy use during winter resulted in reduced body mass post-hibernation (Brischoux et al., 2016). Similarly, winter warming was associated with lower survival rates at emergence in side-blotched lizards (*Uta stansburiana*) (Zani, 2008; Zani et al., 2012). Temperate zone squamates may thus be particularly sensitive to winter climate variability that disrupts dormancy (Moss and MacLeod, 2022). Despite growing evidence that winter warming affects reptile behaviour and physiology, most studies have focused on isolated trait categories – exploring either activity patterns or physiological responses in isolation (Moss and MacLeod, 2022). Hence, the causal links between winter warming, increased activity/arousal and its physiological costs remain largely unexplored.

To clarify links between winter warming, activity levels and associated costs, we experimentally manipulated thermal conditions during winter hibernation in a temperate reptile, the common wall lizard, *Podarcis muralis*. Further, to investigate the effects of multiple winter temperature regimes, we imposed both a constant mild and a fluctuating (5 days cool, 2 days warm) winter warming regime to test their effects on activity and related physiological consequences, including body condition and oxidative stress. Oxidative stress is a key physiological marker of metabolic strain and thermal stress (Bury et al., 2018; Jacobs et al., 2021) and has been used as a proxy for physiological costs of warming in reptiles in response to summer heatwaves (e.g. Han et al., 2024; Li et al., 2021; Zhang et al., 2023). We hypothesised that increased overwinter temperatures, whether constant or fluctuating, would disrupt hibernation in common wall lizards by increasing activity levels during the overwintering period, leading to cumulative physiological consequences. Specifically, we tested three predictions: (i) activity levels of wall lizards will be higher under mild and fluctuating winter regimes compared with a cold winter control; (ii) these winter regimes would result in reduced body condition at emergence; and also (iii) lead to greater oxidative damage, potentially impairing post-hibernation recovery. Although the effects of thermal variability remain poorly understood, we considered two alternative hypotheses regarding the effects of fluctuating winter temperatures. If abrupt temperature shifts are particularly disruptive to overwintering reptiles, we would expect stronger and more negative effects under the fluctuating temperature regime. Alternatively, if overall temperature is more influential for behaviour and physiology (i.e. a threshold effect), then the fluctuating regime, with its regular return to colder phases, would be less disruptive than the constant mild warming. By integrating behavioural and physiological responses under multiple winter warming regimes, this study provides novel insight into the overwintering ecology of a globally expanding reptile and highlights the importance of incorporating behavioural sensitivity, thermal variability and physiological costs into models predicting species responses to climate change.

## MATERIALS AND METHODS

### Ethical approval

Animals were captured with permission from Bournemouth City Council and in partnership with the Amphibian and Reptile Conservation Trust. Experimental procedures and sample collection were conducted in accordance with Home Office regulations under Project Licence PP8703194, held by K.J.M.

### Study species

The common wall lizard, *Podarcis muralis* (Laurenti 1768), is a small lacertid lizard widely distributed across Europe as both a native (Gassert et al., 2013; Jablonski et al., 2019) and more widely still as an introduced species (e.g. Kolenda et al., 2020; Oskyrko et al., 2022; Schulte et al., 2012). The introduced UK populations occur at the northern edge of the species' range (Michaelides et al., 2013, 2015), where it may rely on overwinter torpor as temperatures will frequently dip below the threshold at which foraging and digestion are possible. *Podarcis* lizards hibernate in cool climates (Burke and Ner, 2005; Sakelarieva et al., 2023 preprint), though periods of activity are common when temperatures rise (Foa et al., 1992; Sakelarieva et al., 2023 preprint) Wall lizards probably utilise rock crevices and rock piles as hibernacula (Mackey, 2010). These may provide some buffering from extreme conditions, though evidence suggests crevices buffer cold less effectively than high temperatures (Warner, 2023).

Lizards were captured from an introduced population located in Bournemouth, Dorset (50.72°N, −1.82°W, 10–20 m above sea level), UK. Thirty-nine adult lizards (23 males, 16 females) were caught during the active season in early autumn 2022 using a combination of hand-capture and use of a small ‘lasso’ (a looped filament) attached to an extendable pole. An additional 22 adults (5 males, 17 females) were caught at the same location in early autumn 2023 using the same techniques.

Captured lizards were transported to Bangor University, UK, in thermally insulated containers and plastic terraria within 72 h of capture. Upon arrival at the animal facility at Bangor University, the lizards were housed in plastic terraria (55×35×40 cm) containing a thin layer of soil, various shelters, a basking platform and a water dish. Lizards were individually marked with a non-toxic paint marker on the dorsal surface (neck, mid-back or tail-base) or lateral surface (right or left side) for identification, and were re-marked as necessary when the paint faded. Each terrarium housed 3–5 lizards, with a maximum of 2 males to minimize stress due to intraspecific aggression (Sacchi et al., 2009). No aggressive interactions were ever observed. Heat and natural photoperiod were provided by an 80 W UV basking lamp (9 h light:15 h dark) hung above each terrarium and the main room light (11 h light:13 h dark; average temperature 16±1°C), prior to the experiment. The lizards were provided with water *ad libitum* and fed mealworms, wax worms and/or small crickets 2–3 times per week and given Calci Dust calcium (ProRep, Essex, UK) and Nutrobal vitamin (Vetark, Winchester, UK).

### Experimental design and treatments

To investigate the effects of increased overwinter temperatures on lizard activity, body condition and oxidative stress, individuals were overwintered under one of three experimental thermal treatments. The treatments represent a gradient from cold to mild winter conditions: control, constant mild and fluctuating winter temperatures. The control treatment (4±1°C) simulated typical cold winter conditions in southern UK, where historical records from the Bournemouth airport weather station (metoffice.gov.uk, 1991–2020) indicate that average winter air temperature (December–March) is 3.73°C. The mild winter treatment (8±1°C) simulated warmer winter conditions. The fluctuating winter temperature treatment alternated from cold (5 days of 4±1°C) to mild winter days (2 days of 8±1°C) throughout the experiment, simulating periodic warm spells. These thermal regimes were chosen to represent ecologically relevant variation at the boundary between physiological torpor and intermittent winter activity (Barroso, 2023; While et al., 2015). Moreover, all selected temperatures fall within the natural thermal range experienced by common wall lizards across their UK distribution, where historical minimum and maximum winter air temperatures range from 1.58°C to 11.40°C (metoffice.gov.uk, 1991–2020). Although mild winters in the wild may occasionally exceed 8°C, ethical considerations required us to impose temperature limits to ensure animal welfare, meaning our findings probably represent conservative estimates. We expect that artificial refugia provided in our animal housing provided buffering to a similar level to that which would be expected in rock crevices in natural habitats (Warner, 2023).

We conducted the overwintering experiment over two consecutive winters (each lasting approximately 3.5 months) under consistent protocols and temperature regimes. In year 1, the experiment ran for 104 days (*N*=39 lizards: 23 males, 16 females; captured in autumn 2022). In year 2, the experiment ran for 106 days [*N*=41: 19 survivors from experiment 1 (13 males, 6 females) and 22 newly captured lizards (5 males, 17 females) from autumn 2023]. Mortalities occurred at least 3 months after the overwintering period in year 1 and were probably due to natural ageing, time in captivity or unknown causes. To enhance statistical power and focus on experimental treatment effects, data collected across both winters were pooled for analysis, as experimental conditions remained consistent. Potential inter-annual and hibernation history (whether lizards were first-time or second-time hibernators during the experiment) variation was accounted for by including these terms in our models (see ‘Statistical analysis’, below).

**Fig. 1.**
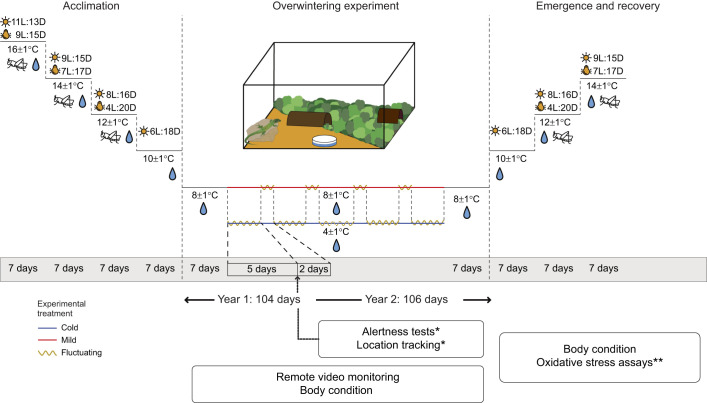
**Experimental design and data collection across the two experimental years.** The diagram illustrates the controlled environmental conditions and data collection timeline throughout the experiment, organised into three main panels: pre-hibernation (acclimation period), hibernation (overwintering experiment) and post-hibernation (emergence and recovery period) of common wall lizards, with corresponding durations in days. The environmental parameters illustrated include photoperiod [main room light indicated by a sun icon, and UV basking light by a light bulb icon, with specific changes in light:dark (L:D) cycles shown in h] and temperature [gradually changed pre- and post-hibernation and over 7 days at the start and towards the end of hibernation, and maintained across three winter temperature treatments: cold treatment (4±1°C; blue line), mild treatment (8±1°C; red line) and fluctuating temperature treatment (5 days at 4°C and 2 days at 8°C; yellow zigzag line)]. Resource availability is shown with a cricket icon for food and a water droplet icon for water. Housing conditions inside the terrarium are illustrated in the centre panel. *Data collected during year 1 only. **Data collected during year 2 only. No symbol for data collected in both years.

We followed and adapted established husbandry guidelines for the safe hibernation of lacertid lizards (Barroso, 2023) to develop our experimental protocol (Fig. 1). Over a ∼30 day acclimation period (beginning in late November), environmental conditions were gradually changed to induce torpor: the photoperiod of both the main room light and UV basking light was progressively reduced (from 9 h light:15 h dark to 6 h light:18 h dark and from 7 h light:17 h dark to 4 h light:20 h dark, respectively) before being switched off. Ambient temperature was also decreased by 2°C every 7 days to reach 8±1°C at the onset of hibernation. Feeding was stopped 7 days prior to the experiment to ensure that no undigested food remained in the lizards' guts, which could rot during hibernation and cause potentially fatal internal infections (Harlow et al., 1976). The lizards were then rehoused in plastic terraria (36×21×16 cm) containing a thin layer of soil, half-covered with sphagnum moss, and equipped with shelters, rocks or stacked slates, and a water dish. Water was provided *ad libitum*. Treatments were assigned randomly, with males and females allocated separately to maintain balanced sex ratios (except for repeated individuals, which were assigned to the same treatment as year 1 to minimise treatment variability; we accounted for this in our models; see ‘Statistical analysis’, below). Each terrarium housed 3–5 individuals. The terraria were placed in temperature-controlled incubators (modified commercial refrigerators) equipped with temperature probe sensors and mini fans to ensure ventilation and prevent ice formation.

During the first 7 days of hibernation, the air temperature was set at 8±1°C across all treatments to allow for a standardized acclimation period. From day 8 onward, the treatments diverged: in the mild winter treatment, the temperature was maintained at 8±1°C throughout the experiment; in the cold winter treatment, the temperature was reduced to 4±1°C; and in the fluctuating winter treatment, temperature alternated between a cool phase (4±1°C, 5 days) and a warm phase (8±1°C, 2 days). Lizards were kept in the dark during hibernation to mimic natural overwintering conditions (e.g. under leaf litter or in crevices). At the end of the experiment, we reversed the pre-hibernation environmental conditions (including a gradual acclimation period of ∼21 days) to induce emergence behaviour in the lizards and progressively started to feed them again.

### Variables collected and parameters measured

#### Activity level

To assess the activity levels of wall lizards during hibernation, we used three complementary methods: alertness tests (year 1), remote video monitoring (year 1 and 2) and location-change tracking (year 1). Both alertness tests and location change tracking were only conducted during the first experimental year (Fig. 1) because of time constraints; this limitation is acknowledged in the Discussion.

Alertness tests were conducted weekly coinciding with routine incubator checks to minimise disturbance. Lizards were assessed for responsiveness to external stimuli in the dark by temporarily removing their terraria from the incubators (lizards remained in place). Visual responsiveness was tested by slowly moving a red light torch (wavelength 620 nm) ∼10 cm in front of their closed eyelids for 5 s. Light at 620 nm provides a detectable visual cue for tetrachromatic reptiles (Osorio, 2019) without the risk of stronger white light disturbing winter torpor. A positive response was recorded if the eyelids opened partially or fully in reaction to the light; no movement was scored as a negative response. Tactile responsiveness was tested by lightly touching the dorsum with a fingertip. A positive response was defined as any visible movement of the head, tail, limbs or eyelids; absence of movement was considered a negative response. All tests were performed on the same weekday, corresponding to the last day of the cool phase, immediately prior to the warm phase of the fluctuating winter treatment, to ensure consistency across treatments.

Remote infrared video monitoring was used to non-invasively assess visible surface activity. Commercially available infrared CCTV weatherproof dome cameras (DTS Digital, Scarborough, North Yorkshire, UK) were installed inside each incubator (3.6 mm fixed lens; 2592×1944 resolution; H.265 codec; .AVI format). Cameras were mounted vertically on the ceiling of the bottom shelf of each incubator ∼30 cm above the terrarium, providing full overhead coverage of the entire arena. Surface activity was quantified using 30 min time-point sampling, based on pilot observations indicating minimal movement between intervals. Although lizards were individually marked, individual-level activity could not be consistently scored from video recordings because markings were often obscured by body position. Therefore, at each sampling point, we recorded: (i) the number of lizards visible outside shelters (reflecting movement related to shelter use) and (ii) the number of lizards with their snouts within 1 cm of the water dish (indicating movement related to water use). Each terrarium was recorded for three consecutive days. Recordings following weekly checks (at the end of the cool phase) were delayed by at least 4 h to allow recovery from handling; no delay was necessary on other days. Four hours was determined sufficient, as lizards typically resumed stationary behaviour or returned to shelters. For the fluctuating winter treatment, activity was recorded during both the cool and warm phases. Recordings were conducted 3 times during the hibernation period (i.e. 3×72 h), in both experimental years (Fig. 1). Consistent lighting conditions and camera placement across incubators ensured comparable visibility.

Location-change tracking was used as a supplementary measure of overall activity accounting for movements not visible on cameras, including movements beneath the substrate or within shelters. Each terrarium was divided into a 5×3 grid (15 cells; 5.6×6.4 cm each). During weekly routine checks in the first experimental year, we recorded the grid cells containing the head, back and tail of each lizard, estimating positions based on the midpoint of each body part. A movement was scored when an individual's body shifted more than two grid cells from its position the previous week, based on the head and body's relative location. This threshold was chosen to capture substantial relocations, providing an indirect measure of arousal, while minimising false positives from minor postural adjustments. For buried or hidden individuals, moss or shelters were briefly lifted to track position using the grid system. All handling and observations were conducted under red light and completed within 10 min per terrarium to minimize disturbance.

#### Body condition

To assess the effects of overwintering temperatures on body condition, we calculated a body condition index (BCI) as the residuals of the linear regression of log_10_-transformed body mass on log_10_-transformed snout–vent length (SVL) (Schulte-Hostedde et al., 2005). This residual-based method is commonly used to evaluate reptile body condition, as it controls for size differences and allows valid comparisons between individuals of varying sizes and ages (Falk et al., 2017; Olsson et al., 2012b; Perry et al., 2024). Because body condition differs markedly between males and females in common wall lizards (Amer et al., 2024; Perry et al., 2024), BCI was calculated separately by sex. For each sex, BCI was calculated from a single log–log regression across three biologically relevant time points: start of the overwintering experiment (S), pre-emergence (E; 1 week before emergence) and post-recovery (R; 3–5 weeks after emergence), observed as a key recovery phase during which feeding and activity resumed regularly (Fig. 1). Residuals were then extracted for each individual at each of these time points. SVL was measured once upon arrival at the animal facilities using a digital calliper (0.1 mm precision). Body mass was recorded monthly throughout the experiment (to 0.01 g precision) to monitor individual health and mass changes.

#### Oxidative stress assays

To assess the physiological costs of increased/fluctuating overwintering temperatures in wall lizards, we collected blood and tissue samples in year 2 to measure three complementary markers of oxidative status (see below). Samples were collected 2 weeks post-emergence to ensure lizards had resumed feeding, as blood collection from recently emerged individuals can be difficult without euthanasia. At the end of the experiment, we sampled from 38 individuals (15 males, 23 females). All sampling was conducted in a randomised order by sex and experimental treatment to avoid confounding effects. Blood was collected from 37 individuals (14 males, 23 females) via the post-orbital sinus, accessed through the corner of the mouth, using heparinised microhaematocrit capillary tubes (Advance Scientific Equipment Pvt. Ltd, Mumbai, India) (Olsson et al., 2018). One individual was excluded following unsuccessful attempts at blood collection to minimise handling stress and potential physiological bias. A total of 20–50 µl blood was collected per individual and immediately centrifuged for approximately 10 min at 2000 ***g***. The resulting plasma (approximately 10–25 µl) was separated and stored at −80°C until oxidative stress assays were performed. Tissue samples were obtained from all 38 individuals by collecting a 1 cm tail clip using sterilized tweezers and scissors. The samples were immediately flash-frozen in liquid nitrogen for approximately 1 min, then stored at −80°C until further analysis.

The oxidative status of the lizards was assessed by determining the level of one antioxidant marker: total antioxidant capacity (TAC); and two oxidative damage markers: malondialdehyde (MDA), indicative of lipid damage, and a combination of three oxidised guanine species (8-hydroxy-2′-deoxyguanosine, 8-hydroxyguanosine and 8-hydroxyguanine) collectively referred to as 8-OHdG, indicative of DNA/RNA damage.

##### TAC concentration determination

TAC in plasma was quantified using a commercially available kit (Antioxidant Assay kit, Cayman Chemical Co., Ann Arbor, MI, USA), following the manufacturer's instructions. We diluted 5 µl of plasma 1:20 with assay buffer to ensure values fell within the assay's sensitivity range. Samples were run in duplicate and results are expressed in mmol l^−1^. The mean intra-assay coefficient of variation (CV) was 2.62%, indicating high precision. Because of limited plasma volume, we could only assay TAC in 23 samples. Absorbance was measured at 750 nm using a Spectramax M2 plate reader (Molecular Devices, Wokingham, UK).

##### MDA concentration determination

MDA concentration in plasma was measured by high-performance liquid chromatography (HPLC) with fluorescence detection using standard techniques (Nussey et al., 2009), with some modifications: 5–10 µl of undiluted plasma was used for the analysis of 37 samples. All chemicals were HPLC grade, and chemical solutions were prepared using ultra-pure water (Milli-Q Synthesis, Millipore, Watford, UK). Sample derivatisation was done in 2 ml capacity screw-top microcentrifuge tubes. To ∼10 µl of sample or standard, 10 µl butylated hydroxytoluene solution (0.05% w/v in 95% ethanol), 80 µl phosphoric acid solution (0.44 mol l^−1^) and 20 µl thiobarbituric acid (TBA) solution (42 mmol l^−1^) were added. The standard was prepared for calibration with 1,1,3,3-tetraethoxypropane (TEP) using a stock solution (5 µmol l^−1^ in 40% ethanol) serially diluted using 40% ethanol. Samples were capped, vortex mixed for 2 s, then heated at 100°C for 1 h on a heat block to allow the formation of MDA–TBA adducts. Samples were then centrifuged at 13,300 ***g*** at 4°C for 1 min, before 80 µl *n*-butanol was added and tubes were vortex mixed for 10 s. Tubes were then centrifuged at 12,000 ***g*** at 4°C for 3 min, before a 50 µl aliquot of the upper (*n*-butanol) phase was collected and transferred to an HPLC vial for analysis.

Samples (20 µl) were injected into an Agilent HPLC system (InfinityLab Solutions, Petaluma, CA, USA) fitted with a 5 µm ODS guard column and a Hewlett-Packard Hypersil 5 µm ODS 100×64.6 mm column maintained at 37°C. The mobile phase was methanol-buffer (40:60, v/v), the buffer being a 50 mmol l^−1^ anhydrous solution of potassium monobasic phosphate at pH 6.8 (adjusted using 5 mol l^−1^ potassium hydroxide solution), running isocratically over 3.5 min at a flow rate of 1 ml min^−1^. Data were collected using a fluorescence detector (Agilent 1200 series) set at 515 nm (excitation) and 553 nm (emission). Results are expressed in μmol l^−1^.

##### 8-OHdG concentration determination

DNA was extracted from tail tissue using a commercially available kit (DNeasy Blood and Tissue kit, Qiagen, Venlo, The Netherlands) following the manufacturer's instructions. The extracted double-stranded DNA (dsDNA) was heat-denatured at 95–100°C for 10 min, then cooled on ice for 5 min to convert into single-stranded DNA (ssDNA). The ssDNA was then digested into single nucleotides using Nuclease P1 (New England Biolabs, Ipswich, MA, USA), incubated at 37°C for 30 min and inactivated at 75°C for 10 min. The solution's pH was then adjusted to 7.5–8.5 with 1 mol l^−1^ Tris before converting the resulting single nucleotides to single nucleosides by adding alkaline phosphatase (Sigma-Aldrich, Burlington, MA, USA) at 1 unit per 100 µg of DNA. The solution was incubated at 37°C for 30 min, then heated at 95°C for 10 min to inactivate the enzyme. The processed samples were analysed using a DNA/RNA Oxidative Damage (High Sensitivity) ELISA kit (Cayman Chemical), following the manufacturer's instructions. Samples were run undiluted in duplicate and results are expressed in pg ml^−1^. A total of 36 samples yielded quantifiable 8-OHdG values and the mean intra-assay CV was 6.42%. Absorbance was measured at 405 nm using a Spectramax M2 plate reader (Molecular Devices, Wokingham, UK).

### Statistical analysis

All statistical analyses were performed using R software version 4.4.2 (http://www.R-project.org/). We used linear models (*lm* function, base *stats* package) for normally distributed continuous data, and generalised linear mixed-effects models (GLMMs) (*glmer* function, *lme4* package; Bates et al., 2015) for repeated measures or count/binary data.

To assess the effect of winter temperature treatment on lizard responsiveness to external stimuli (binary response: positive/negative), we used a GLMM with a binomial error distribution and a logit link function. Treatment (winter temperature regime: cold/mild/fluctuating) was included as a fixed effect, and lizard identity and terrarium identity were included as random effects to account for repeated measures and potential grouping effects. Sex and the interaction treatment×sex were also included as a fixed effects but were removed when they did not improve model fit, as described below.

For visible surface activity, based on remote video observations, we analysed shelter use- and water use-related movements separately using GLMMs with a negative binomial error distribution and a log link function. Daily number of movement events per terrarium was the dependent variable. A movement event was defined as any observed change (increase or decrease) in the number of lizards outside the shelter (for shelter use-related movement) or within 1 cm of the water source (for water use-related movement) between consecutive 30 min time points. Treatment was a fixed effect, and terrarium identity and experimental year (to account for repeated measure) were random effects. Lizard identity was not included as a random term, and sex was not included as a fixed effect, because there was only one measure per terrarium.

Comprehensive activity based on individual location changes was analysed using a GLMM with a Poisson error distribution and log link function. The total number of location changes per individual was the dependent variable. Treatment, sex and their interaction were included as fixed effects, and lizard identity and terrarium identity as random effects.

For body condition (calculated separately for males and females), we first confirmed that there were no baseline differences among treatments or between sexes at the start of hibernation using a linear model (*F*_3,76_=0.49, *P*=0.692). We then used GLMMs with a Gaussian error distribution and identity link function to analyse changes in residual body condition across treatments over two phases: (i) from the start of hibernation (S) to emergence (E), and (ii) from emergence (E) to the end of the recovery phase (R). Fixed effects included treatment, sex, hibernation history (first versus second time hibernators), and the interactions treatment×sex and treatment×hibernation history; terrarium identity and experimental year were included as random effects. Because activity levels may influence energy balance during hibernation, we further tested for associations between activity metrics (visual and tactile responsiveness, location changes) and body condition at emergence and post-recovery using Spearman's rank correlation. As no significant correlations were found (full results in Table S1), activity metrics were not included in the final models.

For oxidative stress markers, we built GLMMs with a Gaussian error distribution and identity link function, including treatment and hibernation history as fixed effects, and terrarium identity as a random effect to account for terrarium grouping. Because physiological condition and oxidative status are often interrelated in vertebrates (Isaksson et al., 2011; López-Castañón et al., 2025; Monaghan et al., 2009), we also examined correlations between each oxidative stress marker (TAC, MDA, 8-OHdG) and body condition of wall lizards. Preliminary Pearson's correlation revealed that TAC level was significantly and positively correlated with body condition post-recovery (*r*_p_=0.49, *P*=0.026). Consequently, to account for this potential confounding effect, BCI post-recovery was included as an explanatory variable in the oxidative stress models. Acknowledging a limitation in our dataset, due to non-overlapping data collection (individual activity metrics recorded in year 1, oxidative stress assays conducted in year 2), activity could not be accounted for in the oxidative stress models. Sex was also not included in the models because of the limited sample size, which precluded robust statistical inference.

For model selection, we started with full models including all relevant fixed effects and their interaction (where biologically justified), and a maximal random effects structure (random intercepts for individual identity, terrarium identity, experimental year, where appropriate). Random effects structures were refined by comparing the models' Akaike information criterion (AIC) and checking for singular fits using the *isSingular* function, retaining more complex structures only when justified. Fixed effects were selected via backward stepwise removal, using likelihood ratio χ^2^ tests (*drop1* function of *stats* package). Assumptions of normality and homogeneity were tested using the Shapiro–Wilk test and for homogeneity of variance using Levene's test (*car* package). GLMM assumptions (e.g. appropriate error distribution and link function, linearity on the link scale) were assessed graphically and using diagnostic tests from the *DHARMa* package (*testDispersion*, *testUniformity* and testOutliers; https://github.com/florianhartig/dharma). Pairwise comparisons were conducted using Tukey-adjusted contrasts (*emmeans* function, *emmeans* package; https://CRAN.R-project.org/package=emmeans). Statistical significance was set to *P*<0.05. We report parameter estimates for significant main effects from the final retained models in the Results; full outputs, including non-significant terms from retained models, are available in supplementary Tables S1–S5.

We did not separate activity-level measurements between the cool and warm phases of the fluctuating temperature treatment for stimuli responsiveness and location change as data were collected during the cool phase, as mentioned above (see ‘Experimental design and treatments’). Moreover, to validate this approach, we further tested for differences in the number of movement events (shelter use- and water use-related) recorded between these phases from remote video monitoring. We used GLMMs with a negative binomial error distribution and a log link function, as described previously, but with phase (cool and warm) as a fixed effect. As we found no significant difference in movement events between the two phases (shelter use: χ_2_^2^=0.64, *P*=0.422; water use: χ_2_^2^=0.01, *P*=0.916; full model results in Table S2), we treated the fluctuating temperature treatment as a single condition in all subsequent analyses.

## RESULTS

### Effects of overwintering temperature on visual and tactile responsiveness

**Fig. 2.**
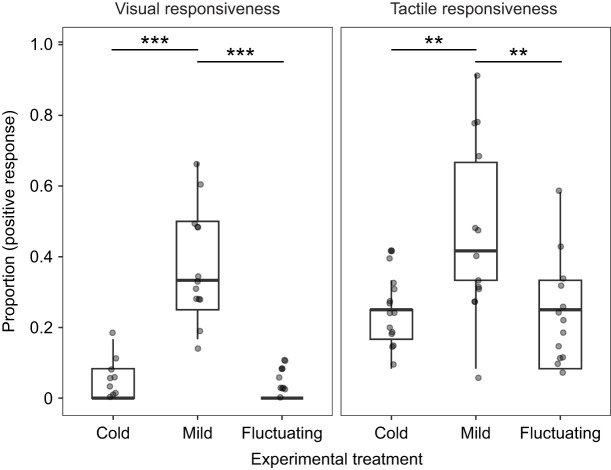
**Effect of temperature treatment on visual and tactile responsiveness of wall lizards during the overwintering experiment.** For the box plots, the top and bottom of the boxes represent the upper and lower quartiles, the horizontal line within the box represents the median and the whiskers represent the 5th and 95th percentiles. Points are individual data records (cold *N*=13, mild *N*=13, fluctuating *N*=13). Asterisks indicate significant differences among treatments (**P<0.005, ***P<0.001) based on Tukey *post hoc* tests of contrasts.

Overwintering temperature significantly affected wall lizard responses to light stimuli (χ_2_^2^=97.49, *P*<0.001; Fig. 2; full model results in Table S3). Lizards from the mild treatment were more likely to respond to light stimuli than those in the cold (odds ratio=15.61, 95% CI [5.41, 45.05], *P*<0.001) or fluctuating temperature treatments (odds ratio=30.04, 95% CI [7.22, 124.93], *P*<0.001), with no difference between the cold and fluctuating temperature treatments (odds ratio=1.92, 95% CI [0.36, 10.35], *P*=0.633). Sex and its interaction with treatment were included in the initial model; the interaction was removed via likelihood ratio tests as it did not improve model fit, while sex had a significant effect (χ_1_^2^=7.84, *P*<0.05), with males being more responsive to light stimuli than females (Table S3).

Similarly, tactile responsiveness differed significantly across temperature treatments (χ_2_^2^=13.81, *P*<0.005; Fig. 2; full model results in Table S3). Lizards from the mild treatment were 3 times more likely to respond to tactile stimuli than those in the cold (odds ratio=3.07, 95% CI [1.44, 6.54], *P*<0.005) and fluctuating temperature treatments (odds ratio=2.99, 95% CI [1.40, 6.38], *P*<0.005). No significant difference in tactile responsiveness was found between the cold and fluctuating winter temperatures (odds ratio=0.98, 95% CI [0.44, 2.15], *P*=0.997). Sex and its interaction with treatment did not improve model fit and were removed from the final model.

### Effects of overwintering temperature on visible surface and comprehensive activity

**Fig. 3.**
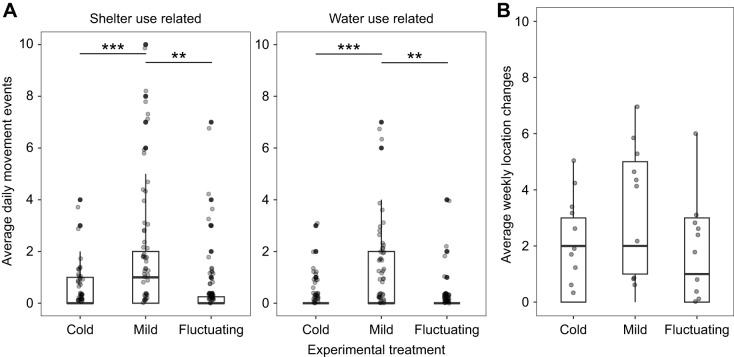
**Effect of temperature treatment on metrics of overwintering movement activities in wall lizards.** (A) Average daily movement events of wall lizards related to shelter and water use during the overwintering experiment across three temperature treatments (cold, mild and fluctuating). For the box plots, the top and bottom of the boxes represent the upper and lower quartiles, the horizontal line within the box represents the median and the whiskers represent the 5th and 95th percentiles. Points are individual data records combined across two experimental years (cold *N*=21, mild *N*=20, fluctuating *N*=20). Asterisks indicate significant differences among treatments (**P<0.005, ***P<0.001) based on Tukey *post hoc* tests of contrasts. (B) Average weekly location changes of wall lizards inside terraria during the overwintering experiment across three temperature treatments (cold, mild and fluctuating). Box plots as in A. Points are individual data records (cold *N*=13, mild *N*=13, fluctuating *N*=13).

Movements were observed across all temperature treatments, including movements in and out of shelters, towards the water source, and both above and underground activity (Fig. 3). Weekly observations of individual location changes revealed no significant difference in movement frequency across treatments (χ_2_^2^=2.27, *P*=0.322; Fig. 3B; full model results in Table S3). However, treatment significantly affected visible surface activity, as measured by remote video monitoring, including shelter use (χ_2_^2^=27.22, *P*<0.001) and water use (χ_2_^2^=18.71, *P*<0.001) (Fig. 3A; full model results in Table S3). Sex and its interaction with treatment did not improve model fit and were removed.

The average daily number of shelter-related movements per terrarium was over 4 times higher in the mild treatment than in the cold treatment (odds ratio=4.504, 95% CI [2.05, 9.90], *P*<0.001), and over 3 times higher than in the fluctuating temperature treatment (odds ratio=3.39, 95% CI [1.61, 7.11], *P*<0.005). Similarly, lizards in the mild treatment were significantly more likely to travel towards the water source than those in the cold (odds ratio=4.37, 95% CI [1.88, 10.20], *P*<0.001) or fluctuating temperature treatments (odds ratio=3.40, 95% CI [1.44, 8.028], *P*<0.005). No significant differences were found between the cold and fluctuating winter temperatures for either shelter use (odds ratio=0.75, 95% CI [0.32, 1.77], *P*=0.712) or water use (odds ratio=0.78, 95% CI [0.30, 1.987], *P*=0.804).

### Changes in body condition post-hibernation and post-recovery in response to overwintering temperature

**Fig. 4.**
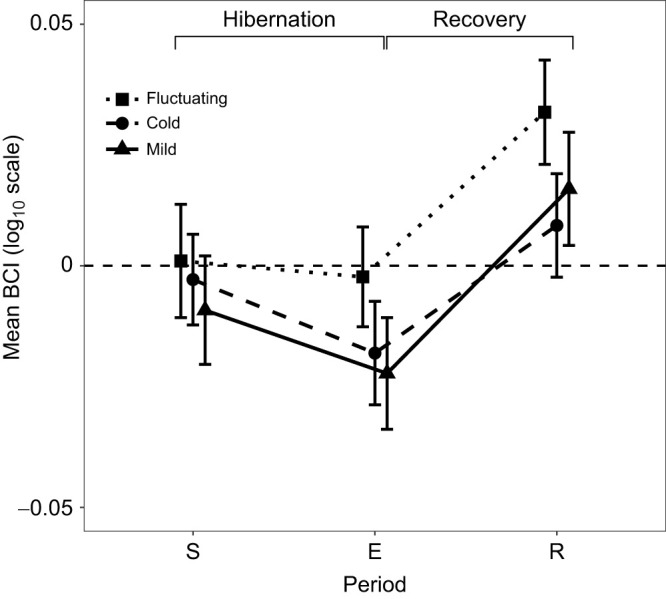
**Change in body condition of wall lizards during hibernation and the key recovery period.** Mean±s.e.m. body condition [residuals±s.e.m. of the linear regression of log_10_-transformed body mass on log_10_-transformed snout–vent length (SVL), calculated separately by sex, no units] is shown for lizards exposed to cold (circles, dashed line), mild (triangles, solid line) and fluctuating (squares, dotted line) overwintering temperatures. Time points are: S, start of hibernation; E, pre-emergence (1 week before emergence); and R, post-recovery (3–5 weeks after emergence). The horizontal dashed line at zero indicates no change in body condition from initial measurements. Sample sizes were *N*=21 cold treatment, *N*=20 mild treatment and *N*=20 fluctuating temperature treatment for time points S and E. For time point R, sample sizes were *N*=19 cold treatment, *N*=20 mild treatment and *N*=19 fluctuating temperature treatment.

At the onset of the overwintering experiment, body condition of wall lizards (residuals of the linear regression of log_10_-transformed body mass on log_10_-transformed SVL, calculated separately by sex) did not differ among treatments (*F*_2,76_=0.24, *P*=0.788; Table 1). Unsurprisingly, body condition declined in all treatments by the end of hibernation both in males (mean±s.e.m.: −0.017±0.003) and females (−0.003±0.005), with no significant treatment effect (*F*_2,73_=4.20, *P*=0.122; Fig. 4, Table 1). However, second-time hibernators showed a significantly greater decline in body condition at the end of hibernation compared with first-timers (*F*_1,73_=5.10, *P*<0.05). Sex and its interaction with treatment were included in initial models for both the hibernation and recovery phases but did not improve model fit following likelihood ratio tests and were therefore removed from final models.

**Table 1.** Summary statistics of the physiological parameters (body condition index and oxidative status indices) measured across treatments for individual common wall lizards

		Treatment		
Parameters	Sex	Cold	Mild	Fluctuating
BCI baseline (residuals)	M	−0.010±0.012 (15)	−0.006±0.013 (13)	0.026±0.013 (13)
	F	0.006±0.014 (13)	−0.013±0.019 (12)	−0.022±0.017 (14)
BCI post-hibernation (residuals)	M	−0.019±0.006 (15)	−0.021±0.005 (13)	−0.011±0.005 (13)
	F	−0.010±0.012 (13)	−0.004±0.006 (12)	0.004±0.010 (14)
BCI post-recovery (residuals)	M	0.023±0.011 (14)	0.024±0.008 (11)	0.023±0.008 (13)
	F	0.016±0.011 (12)	0.049±0.010 (12)	0.036±0.013 (13)
TAC (mmol l^−1^)		1.595±0.245 (10)	1.944±0.198 (6)	1.700±0.559 (7)
MDA (µMol)		15.062±1.640 (14)	18.234±1.606 (10)	16.187±1.795 (13)
8-OhdG (pg ml^−1^)		381.406±49.753 (14)	572.709±63.506 (10)	467.920±54.719 (12)

Data are means±s.e.m. (*N* sample size) for the different treatment conditions: cold, mild and fluctuating winter temperatures. Body condition index (BCI) was calculated as the residuals from sex-specific linear regressions of log_10_-transformed body mass on log_10_-transformed snout–vent length (SVL) and is shown for males (M) and females (F). TAC, total antioxidant capacity; MDA, malondialdehyde; 8-OHdG, oxidised guanine species.

During the recovery phase (3–5 weeks following hibernation), body condition improved across all treatments both in males (mean±s.e.m.: 0.024±0.005) and females (0.033±0.007), again with no significant differences among treatments (*F*_2,71_=0.88, *P*=0.417; Fig. 4, Table 1). Recovery was significantly lower in second-time hibernators (*F*_1,71_=13.03, *P*<0.001), consistent across treatments. Despite higher winter activity observed in the mild treatment (see previous section), this did not translate into greater body condition loss or impaired recovery (see also Table S1 for results of correlation between activity levels and body condition index), suggesting compensatory regulation of energy balance. Full model results for BCI are shown in Table S4.

### Effect of overwintering temperature on oxidative status indices

**Fig. 5.**
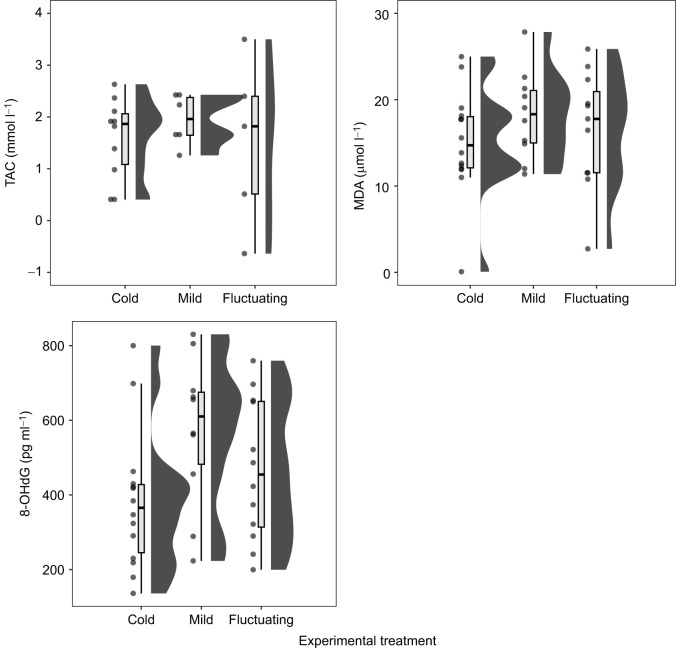
**Effect of temperature treatment on oxidative indices in wall lizards.** Combined dot, box and half violin plots illustrate the concentration of total antioxidant capacity (TAC), malondialdehyde (MDA) and a combination of three oxidised guanine species (8-OHdG) in wall lizards from the three temperature treatments (cold, mild and fluctuating). For the box plots, the top and bottom of the boxes represent the upper and lower quartiles, the horizontal line within the box represents the median and the whiskers represent the 5th and 95th percentiles. Points are individual data records (TAC: cold *N*=10, mild *N*=6, fluctuating *N*=7; MDA: cold *N*=14, mild *N*=10, fluctuating *N*=13; 8-OHdG: cold *N*=14, mild *N*=10, fluctuating *N*=12). The half violin plots display the kernel probability density of the data, with wider sections indicating higher data density.

TAC and MDA concentration did not vary significantly across treatments (TAC: *F*_2,8.1_=0.24, *P*=0.789; MDA: *F*_2,17_=0.42, *P*=0.663; Fig. 5, Table 1), indicating that neither antioxidant capacity nor lipid peroxidation was significantly affected by overwintering temperature. However, TAC was significantly and positively associated with body condition post-recovery (*F*_1,13.6_=7.22, *P*<0.05), suggesting that individuals recovering better following hibernation had higher antioxidant levels.

In contrast, levels of DNA damage – quantified as the concentration of three combined oxidised guanine species (8-hydroxy-2′-deoxyguanosine, 8-hydroxyguanosine and 8-hydroxyguanine, referred to as 8-OHdG) – showed a trend towards a treatment effect (*F*_2,32_=2.99, *P*=0.050; Fig. 5). Although *post hoc* pairwise comparisons did not reach significance after Tukey correction – probably because of limited statistical power – the contrast between cold and mild winter treatments approached significance (β=−191.3 pg ml^−1^; 95% CI: [−384.3, 1.73]; *P*=0.052; Table 1), suggesting a potential increase in DNA oxidative damage under warmer winter conditions. The difference between cold and fluctuating temperature treatments was smaller (β=−65.7 pg ml^−1^; 95% CI: [−253.6, 122.10], *P*=0.669), while fluctuating temperature and mild treatments differed by −125.6 pg ml^−1^ (95% CI: [−329.26, 78.1]; *P*=0.298). None of the oxidative stress markers differed between first- and second-time hibernators (full model results are provided in Table S5).

## DISCUSSION

Winter warming is becoming an important focus in climate change research, as the cold, inactive season has historically been overlooked despite often experiencing greater environmental change than the warm, active season (Intergovernmental Panel on Climate Change, 2014; Vose et al., 2005). Ectotherms, among the most vulnerable organisms to climate change (Wild et al., 2025), provide valuable models for understanding overwintering responses to warming. We experimentally examined behavioural and physiological responses to winter warming in a temperate reptile, the common wall lizard (*Podarcis muralis*), under ecologically relevant overwintering conditions. To our knowledge, this is the first study to simultaneously assess changes in winter activity and subsequent physiological consequences (body condition and oxidative damage) in a reptile under controlled winter conditions, and one of the few to examine multiple winter warming regimes concurrently.

As expected, consistently higher winter temperatures led to increased activity during hibernation. These behavioural changes were not accompanied by major physiological shifts in terms of body condition or most oxidative status indices. However, notably, we observed a trend toward increased DNA damage under mild winter temperatures, highlighting a potentially overlooked physiological vulnerability during dormancy that merits further investigation in reptiles. Given that natural winter conditions rarely follow a uniformly warm pattern, our inclusion of a fluctuating regime adds ecological realism. Interestingly, we did not find effects on measures of activity, condition or oxidative stress of a fluctuating winter warming regime, where 2 out of every 7 days were ‘mild’. Our results suggest that *P*. *muralis* is resilient to moderate winter warming, although more consequential effects are possible. We explore this finding and alternative explanations below, while acknowledging the limitations of our experimental approach, which may lead to conservative estimates of the true effects in more complex natural settings.

### Mild temperature increased winter activity in common wall lizards

Activity levels in common wall lizards remained low under cold and fluctuating winter temperatures but increased significantly under mild conditions. Although winter activity in reptiles is understudied relative to that during the active season (but see Nordberg and Cobb, 2016, 2017; Sperry et al., 2010; Sperry and Weatherhead, 2012), our findings align with growing evidence that milder winters can promote mid-winter arousal and movement in temperate ectotherms (Nordberg and Cobb, 2016, 2017; Turner and Maclean, 2022). Notably, wall lizards in many natural populations remain active year-round, with individuals observed basking on sunny days even in mid-winter (e.g. Sakelarieva et al., 2023 preprint), suggesting that brief activity bouts may be more widespread than previously appreciated. Unlike mammals that enter true hibernation (i.e. multiday torpor with markedly lowered body temperature, reduced physiological functions and complete inactivity), reptiles experience interrupted dormancy, marked by brief, temperature-dependent periods of arousal (Gregory, 1982). The increased movement and alertness observed in the mild treatment (regardless of sex, though males were generally more alert) suggest that warmer temperatures may induce a lighter dormancy state, increasing lizards' responsiveness to ambient cues. Informal observations during routine checks suggested tongue-flicking behaviour, a temperature-sensitive response (Stevenson et al., 1985), was exclusively noted in the mild treatment group, which aligns with increased responsiveness. This heightened reactivity could be adaptive in natural settings; for example, by improving predator avoidance or escape during vulnerable periods such as dormancy (Stawski et al., 2015).

While winter activity is often linked to opportunities for basking in sunny conditions (Nordberg and Cobb, 2016), our experiment was conducted entirely in the dark, suggesting that temperature alone, independent of photoperiod, can trigger arousal during dormancy. Furthermore, activity patterns did not seem randomly distributed across time and exhibited temporal clustering (Fig. S1), hinting at a persistent residual circadian influence that may shape arousal timing and energy expenditure in overwintering reptiles. Although increased winter activity could be beneficial in environments where food is available, we did not assess the energetic costs associated with this elevated activity. In the wild, such costs could be considerable where food is scarce – a scenario increasingly common with climate shifts affecting seasonal trophic synchrony (Damien and Tougeron, 2019; Forrest, 2016). Even when food is available, lower temperatures are likely to reduce food intake and increase digestive passage time, limiting potential gains from increased foraging. Digestion efficiency itself appears relatively unaffected by temperature in reptiles broadly (Wehrle and German, 2023) and in common wall lizards specifically (Parks et al., 2025), although further research is needed.

Interestingly, the variable temperature regime (fluctuating winter treatment) did not lead to a significant overall increase in activity compared with the cold winter treatment. Furthermore, even within the fluctuating temperature phases (cool and warm phases), movement rates during the warm periods did not significantly increase. This suggests that there is a threshold duration or intensity below which periods of milder temperatures are not sufficient to elicit a full or sustained arousal response from dormancy. This dampened sensitivity to temperature cues, within a certain range or of less than a certain duration, could represent an adaptive strategy for common wall lizards with variable temperatures in temperate regions. Where environments are characterised by natural, short-term thermal variability during winter, maintaining dormancy despite minor fluctuations would conserve vital energy reserves and minimise exposure to predation or starvation when food is not available (e.g. Pretzlaff et al., 2021). In contrast, more prolonged or extreme warming events, such as mid-winter heatwaves, might surpass this threshold and trigger more behavioural responses, with associated physiological costs. Despite its ecological relevance, thermal variability has rarely been incorporated into experimental overwintering studies (Moss and MacLeod, 2022). Our findings highlight the need for more nuanced experiments that account for thermal fluctuations to better understand how animals respond to realistic climate warming scenarios, especially as variability and thermal extremes become more common under climate change (Intergovernmental Panel on Climate Change, 2023).

### Increased winter activity did not result in major physiological consequences in common wall lizards

In contrast to previous studies reporting declining reptile body condition with winter warming, probably due to greater metabolic demands (Brischoux et al., 2016; Muir et al., 2013), we found no differences in body condition at emergence and post-recovery across our three winter temperature treatments. Moreover, we found no evidence of greater body condition decline or impaired recovery associated with higher winter activity. The discrepancy between prior findings and ours may be due to differences in temperature regime: in previous studies, winter temperature increases were more pronounced (≥ 6°C), whereas our experiments simulated more moderate variations (≤ 4°C), which may not have been strong enough to induce significant changes in body condition.

Alternatively, lizards in our study may have buffered these effects through behavioural adjustments. Movements towards the water dish were significantly higher under mild temperatures, which is relevant given that hydration is critical for reptile survival (Munsey, 1972), and dehydration can exacerbate heat stress (Dezetter et al., 2022; Dupoué et al., 2018). Higher water intake could have helped offset the energetic costs associated with increased mild winter activity and/or allowed individuals to better maintain water balance, thereby sustaining body condition throughout hibernation and the recovery period*.* Our assessment of body condition was also based solely on body mass changes, which may not fully reflect physiological state (e.g. Warner et al., 2016; Zani et al., 2012). More precise metrics, such as fat content in the liver, muscle or abdominal vesicles (e.g. Church, 1962), would provide a better measure of energy reserves. Further investigation, including metabolic rate measurements (e.g. Kelm and von Helversen, 2007), would help clarify the energetic costs of winter warming.

Beyond temperature treatment effects, individual hibernation history during the experiment influenced body condition outcomes: second-time laboratory hibernators exhibited greater body condition decline during hibernation, followed by reduced recovery compared with first-time laboratory hibernators. Although animals were considered naive within the context of the experimental design, they were caught as adults and probably experienced natural hibernation before capture; such prior experience cannot be excluded as a contributing factor to physiological responses. As the observed body condition decline was consistent across treatments, it may reflect cumulative physiological costs of repeated hibernation or age-related declines in metabolic performance (Speakman, 2005). Although age was not directly measured, the short lifespan of *P*. *muralis* in general (Castanet and Roche, 1981; Vollono and Guarino, 2002) suggests that even modest age differences may influence overwintering outcomes. Alternatively, time in captivity (which here unavoidably covaries with previous experimental overwintering experience) may alter physiological processes (e.g. Navas and Gomes, 2001) and could contribute to variation in overwintering responses. These findings highlight the importance of longitudinal or repeated-exposure studies, as physiological responses to environmental stressors may vary with age, prior experience, time in captivity or cumulative exposure – factors that may not be captured in single-experiment designs.

Lipid peroxidation, inferred from MDA concentration, did not differ significantly between treatments. This suggests that winter warming did not increase lipid damage in *P*. *muralis*, potentially indicating a limited sensitivity of this species to lipid peroxidation. Although no previous studies have examined MDA responses to winter warming in reptiles, research on summer heatwaves has provided mixed results, highlighting species-specific patterns in oxidative damage. For example, MDA levels increased following heat stress in desert lizards (*Eremias multiocellata*) (Han et al., 2020), but decreased in arid toad-headed agamas (*Phrynocephalus przewalskii*) under moderate heatwaves (Han et al., 2024). Meanwhile, no effect of heatwaves on MDA levels was observed in turtles (Li et al., 2021; Zhang et al., 2019). In our study, the absence of a treatment effect may reflect a true lack of increased lipid peroxidation under winter warming. Alternatively, lipid peroxidation could have occurred during hibernation but remained undetected post-hibernation owing to efficient antioxidant defences. Lipid-targeted antioxidants, such as vitamin E, β-carotene, glutathione or glutathione peroxidase, may have mitigated oxidative damage and maintained stable MDA levels (Musalmah et al., 2002). This would suggest that lipid damage in this context is more likely to be a response to acute heat stress rather than a direct consequence of increased winter activity.

TAC also did not differ across treatments, indicating a stable overall antioxidant response. Because the TAC assay reflects combined enzymatic and non-enzymatic activity, treatment-specific changes in individual components [e.g. elevated β-carotene and/or glutathione peroxidase alongside reduced superoxide dismutase (SOD)] could mask shifts in specific antioxidant pathways. For example, elevated lipid-targeted antioxidants may limit MDA accumulation, while reduced SOD activity could contribute to increased DNA damage, as previously reported in reptiles (Olsson et al., 2012a,b, 2018). Such compensatory shifts result in no net change in TAC and explain why lipid damage remained low while oxidative DNA damage was more responsive. However, as we did not measure individual antioxidant components, this hypothesis remains speculative. Importantly, we found that TAC concentrations were positively correlated with body condition post-recovery, suggesting that individuals with stronger antioxidant defences were better able to regain mass post-hibernation. This supports the idea that antioxidant capacity may contribute to overwintering resilience (e.g. Wu and Storey, 2016), even if not directly shaped by winter temperature. Nonetheless, the lack of TAC measurements pre-hibernation limits our ability to determine whether the observed levels post-hibernation reflect strong or weak antioxidant defences. Without a baseline for comparison, it is unclear whether overwintering had a protective or detrimental effect on the lizards' antioxidant capacity.

Although the effect of winter temperature on 8-OHdG levels, an indicator of DNA damage, did not reach conventional statistical significance (treatment effect: *P*=0.050; cold versus mild *post hoc* comparison: *P*=0.052), the observed pattern suggests increased DNA oxidation under milder winter conditions. Moreover, based on estimated marginal means from our models, lizards in the mild treatment showed ∼50% higher levels of DNA oxidation than those in the cold treatment (mean±s.e.m: 573±64 versus 381±50 pg ml^−1^). This trend may reflect a biologically relevant effect that merits consideration beyond strict *P*-value thresholds (Wasserstein et al., 2019). Comparable magnitude changes in 8-OHdG have been reported in an arid-zone lizard (*Pogona vitticeps*) exposed to ecological stressors such as sublethal pesticide exposure (≥45% change; Contador-Kelsall et al., 2023). Experimental increases in DNA damage have also been linked to fitness-related trait loss, including erosion of sexually selected colouration in painted dragon lizards (*Ctenophorus pictus*) (Olsson et al., 2012a).

Interestingly, our findings contrast with evidence that an increase in temperature enhances DNA repair efficiency in ectotherms (MacFadyen et al., 2004; Morison et al., 2020), suggesting that mild winter warming may not support efficient repair during hibernation, thereby allowing oxidative damage to accumulate. This is particularly noteworthy given the long-term fitness effects of DNA damage, including mutation risk, impaired cellular function and compromised tissue integrity over time (López-Otín et al., 2013). Previous research on overwintering reptiles has focused primarily on the effects of supercooling and freezing, followed by thawing, on oxidative stress; notably, Voituron et al. (2006) found no significant effect of extremely low winter temperatures on DNA damage in reptiles, suggesting that cold-induced stress may not always result in oxidative DNA modifications. While no studies have examined the effects of mild or warm winter temperatures on DNA damage markers in reptiles, research in fish supports our findings. A study in sticklebacks reported higher oxidative stress levels in fish exposed to warmer conditions, highlighting the potential for increased oxidative challenge under elevated temperatures (Kim et al., 2019). Given the cautious nature of our experimental temperature increase and our relatively low sample size, our findings probably represent conservative estimates. In natural settings, winter air temperatures in Bournemouth, UK, where the lizards were sampled, occasionally exceed our experimental conditions (metoffice.gov.uk, 1991–2020), with average maximum temperatures of 11.4°C; under such conditions, we would probably observe greater effects in this oxidative stress metric, making the DNA damage marker a good target for future studies in wild reptiles.

The limited physiological consequences observed in this study may be explained by several factors. First, hormetic effects – non-linear relationships between stressors and fitness (Calabrese, 2013) which are particularly relevant in acclimation experiments – may have played a role. Our experiment lasted for 3.5 months, and previous exposure to mild temperatures during the initial weeks of hibernation may have primed individuals to functionally mitigate oxidative stress in the later stages of the experiment (Costantini et al., 2012). However, this remains speculative, as confirming such an effect would require sampling before, during and after the experiment – an approach that is logistically challenging and may itself introduce additional stress to the animals.

Second, organisms adapted to variable environmental conditions often exhibit physiological mechanisms that enable them to cope with temperature fluctuations and mitigate oxidative stress (Ritchie and Friesen, 2022). Common wall lizards are a resilient species with a broad thermal tolerance, allowing them to expand beyond their typical thermal range (Jablonski et al., 2019; Kolenda et al., 2020), including colonizing regions such as the UK and Canada. They have demonstrated rapid physiological plasticity in response to cold temperatures (Haro et al., 2023) and possess high ecological plasticity (Capula et al., 1993; Pérez i de Lanuza and Carretero, 2018; Žagar, 2016), which may be driven by their genetic background (Schulte et al., 2012). *Podarcis muralis* also occurs across a wide range of climatic zones, including Mediterranean regions where warmer winters are natural. As such, the thermal conditions encountered in this experiment are not far outside the norm for this species and may not have posed a strong enough challenge to trigger substantial physiological disruptions.

Third, our results may be limited by the relatively small sample size for each oxidative stress marker, reducing statistical power for robust analyses. Additionally, oxidative stress markers can vary considerably between different tissues (Madeira et al., 2016; Vinagre et al., 2014). When oxidative damage occurs in the liver, it can impair blood detoxification and glycogen storage (Oettl et al., 2013; Zhu et al., 2012). However, oxidative damage in brain tissue has been linked to reduced cognitive performance, including changes in behaviour and slow response times (Forster et al., 1996; Mariani et al., 2005). To avoid the need to euthanise animals, we limited our study to blood serum and tail tissue samples, which may not fully capture the systemic effects of oxidative stress. Future studies should consider measuring reactive oxygen species levels in specific organs, such as the spleen, brain or liver, to provide a more comprehensive understanding of the physiological impacts of winter warming.

Finally, our findings are limited by the absence of other key fitness-related metrics, such as reproductive output and long-term survival, which are crucial for assessing the broader ecological consequences of increased overwintering temperatures (Moss and MacLeod, 2022). Moreover, additional physiological markers, such as telomere length and hormonal stress indicators, could provide further insight into the long-term stress responses of wall lizards under mild winter conditions. As climate change increases the frequency and intensity of winter thermal fluctuations (Vose et al., 2005), it is now critical to explore how short-term winter heatwave events – not just consistently mild warming – challenge overwintering ectotherms, particularly those from more extreme high-latitude environments. Together, our findings highlight the importance of integrating behavioural sensitivity, thermal variability and hidden physiological costs into models predicting species responses to climate change.

## Supplementary Material

10.1242/jexbio.251440_sup1 Supplementary information
