# Supplementary material for: Experimental winter warming increases activity with signs of potential DNA damage in common wall lizards
Source: J Exp Biol. 2025 Nov 21;228(22):jeb251440. doi: 10.1242/jeb.251440 (PMC12690454; doi:10.1242/jeb.251440)
Supplement: Supplementary information [file jexbio-228-251440-s1.pdf]

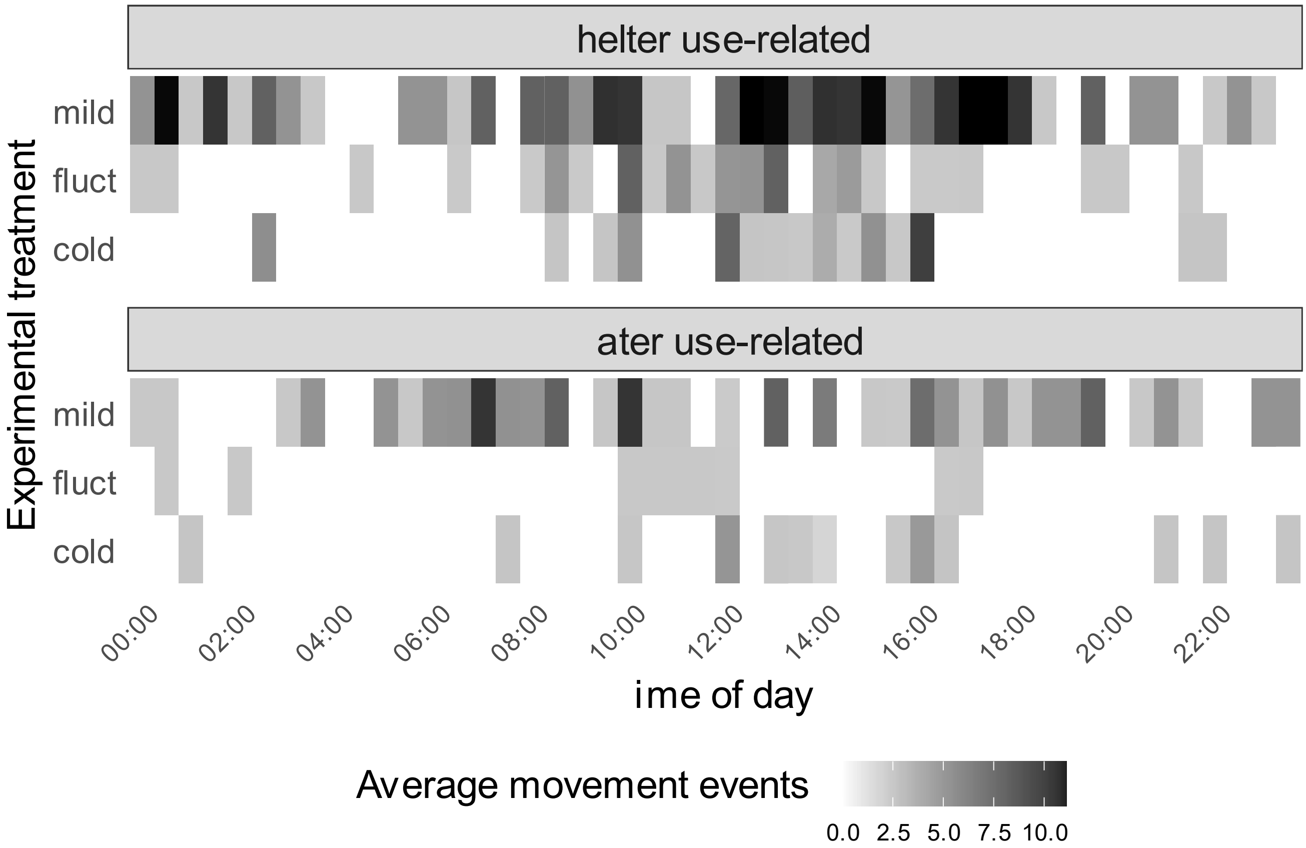


**Fig. S1. Hourly movement events related to shelter and water use across winter temperature treatments.** Heatmap showing the average hourly movement events of wall lizards during the overwintering experiment under cold, fluctuating (labelled “fluct” in the figure), and mild winter temperature treatments. Data were combined across two experimental years (cold *N*=21; mild *N*=20; fluctuating *N*=20).

**Table S1. Correlation coefficients and p-values for relationships between activity metrics, body condition, and oxidative stress markers.**

| Variables | BCI emergence | | | BCI recovery | | |
|---|---|---|---|---|---|---|
| | *N* | *r* | *P* | *N* | *r* | *P* |
| Visual responsiveness | 39 | 0.06 | 0.699 | 38 | -0.19 | 0.252 |
| Tactile responsiveness | 39 | -0.01 | 0.960 | 38 | -0.13 | 0.453 |
| Location change | 39 | -0.27 | 0.096 | 38 | -0.10 | 0.536 |
| **Total antioxidant capacity** | 23 | -0.20 | 0.392 | 23 | 0.49 | **0.026** |
| Malondialdehyde | 37 | 0.05 | 0.786 | 37 | 0.26 | 0.139 |
| Oxidised guanine species | 36 | 0.05 | 0.751 | 36 | 0.20 | 0.248 |

Body Condition Index (BCI) was calculated as the residuals from sex-specific linear regressions of $\log_{10}$-transformed body mass on $\log_{10}$-transformed snout–vent length (SVL). Spearman's rank correlation was used for activity metrics (visual responsiveness, tactile responsiveness, and location changes) and Pearson's correlation for oxidative stress markers. Significant p-values (<0.05) are shown in bold.

**Table S2. Generalized Linear Mixed Model summary for daily movement events related to shelter and water use across the two phases of the fluctuating temperature treatment.**

| Response variable | *N* | Treatment | Odds ratio | 95% CI | *P* |
|---|---|---|---|---|---|
| Shelter use-related movement | 61 | Intercept | 0.44 | 0.21, 0.88 | **0.021** |
| | | Warm phase | 1.66 | 0.47, 5.84 | 0.473 |
| Water use-related movement | 61 | Intercept | 0.002 | 0.00, 6.17 | 0.127 |
| | | Warm phase | 1.13 | 0.11, 11.70 | 0.916 |

Cool phase (intercept): 4±1°C, 5 days; warm phase: 8±1°C, 2 days. Significant p-values (<0.05) are shown in bold.

Journal of Experimental Biology • Supplementary information

**Table S3. Effect of treatments on metrics of overwintering activities of wall lizards during the overwintering experiment: GLMMs and post-hoc results.**

| Response variable | *N* | Fixed effects | Odds ratio | *t* | *P* |
|---|---|---|---|---|---|
| Visual responsiveness | 39 | **(Intercept)** | 0.02 | -7.83 | **<0.001** |
| | | **Treatment** | | | |
| | | Mild | 15.61 | 6.08 | **<0.001** |
| | | Fluctuating | 0.52 | -0.91 | 0.362 |
| | | **Sex** | | | |
| | | Male | 2.45 | 2.71 | **0.007** |
| | Interaction removed: treatment x sex ($\chi_2^2$=0.15, *P*=0.930) | | | | |
| Tactile responsiveness | 39 | **Treatment** | | | **<0.005** |
| | | Intercept | 0.28 | -5.24 | **<0.001** |
| | | Mild | 3.06 | 3.47 | **<0.001** |
| | | Fluctuating | 1.03 | 0.07 | 0.941 |
| | Fixed effects removed: sex ($\chi_1^2$=0.37, *P*=0.124); treatment x sex ($\chi_2^2$=2.33, *P*=0.312) | | | | |
| Shelter use-related movement | 61 | **Treatment** | | | **<0.001** |
| | | Intercept | 0.39 | -3.52 | **<0.005** |
| | | Mild | 4.51 | 4.49 | **<0.001** |
| | | Fluctuating | 1.33 | 0.79 | 0.432 |
| Water use-related movement | 61 | **Treatment** | | | **<0.001** |
| | | Intercept | 0.18 | -3.47 | **<0.001** |
| | | Mild | 4.71 | 3.21 | **<0.005** |
| | | Fluctuating | 0.38 | -1.22 | 0.222 |
| Location change | 39 | **Treatment** | | | 0.322 |
| | | Intercept | 0.11 | -7.02 | **<0.001** |
| | | Mild | 1.47 | 0.97 | 0.332 |
| | | Fluctuating | 0.80 | -0.53 | 0.596 |
| | Fixed effects removed: sex ($\chi_1^2$=0.12, *P*=0.731); treatment x sex ($\chi_2^2$=0.71, *P*=0.702) | | | | |

| Response variable | Treatment comparison | Odds ratio | 95% CI | *P* |
|---|---|---|---|---|
| Visual responsiveness | Cold – Fluctuating | 1.92 | 0.36, 10.35 | 0.632 |
| | **Mild – Cold** | 15.61 | 5.41, 45.05 | **<0.001** |
| | **Mild – Fluctuating** | 30.04 | 7.22, 124.93 | **<0.001** |
| Tactile responsiveness | Cold – Fluctuating | 0.98 | 0.44, 2.15 | 0.997 |
| | **Mild – Cold** | 3.07 | 1.44, 6.54 | **<0.005** |
| | **Mild – Fluctuating** | 2.99 | 1.40, 6.38 | **<0.005** |
| Shelter use-related movement | Cold – Fluctuating | 0.75 | 0.32, 1.77 | 0.712 |
| | **Mild – Cold** | 4.50 | 2.05, 9.90 | **<0.001** |
| | **Mild – Fluctuating** | 3.39 | 1.61, 7.11 | **<0.001** |
| Water use-related movement | Cold – Fluctuating | 0.78 | 0.30, 1.99 | 0.804 |
| | **Mild – Cold** | 4.37 | 1.88, 10.20 | **<0.001** |
| | **Mild – Fluctuating** | 3.40 | 1.44, 8.03 | **<0.005** |
| Location change | Cold – Fluctuating | 1.26 | 0.46, 3.45 | 0.857 |
| | Mild – Cold | 1.47 | 0.58, 3.76 | 0.596 |
| | Mild – Fluctuating | 1.85 | 0.70, 4.88 | 0.297 |

Pairwise comparisons between treatments were performed using *emmeans* with Tukey's method for p-value adjustment. Significant p-values (<0.05) are shown in bold.

**Table S4. Effect of treatments on body condition of wall lizards at emergence and after the recovery phase: GLMMs and post-hoc results.**

| Response variable | *N* | Fixed effects | Estimates | *t* | *P* |
|---|---|---|---|---|---|
| BCI emergence | 61 | **(Intercept)** | -0.012 | -0.84 | 0.653 |
| | | **Treatment** | | | |
| | | Mild | 0.003 | 0.52 | 0.604 |
| | | Fluctuating | 0.013 | 1.99 | 0.054 |
| | | **Hibernation history** | | | |
| | | Second-time | -0.017 | -2.26 | **0.025** |
| | | Fixed effects removed: sex ($F_{1,73}$=0.75, $P$=0.385); treatment x sex ($F_{2,70}$=0.07, $P$=0.964); treatment x hibernation history ($F_{2,72}$=0.93, $P$=0.627) | | | |
| BCI recovery | 58 | **(Intercept)** | 0.029 | 4.00 | **<0.001** |
| | | **Treatment** | | | |
| | | Mild | 0.012 | 1.24 | 0.220 |
| | | Fluctuating | 0.010 | 1.02 | 0.310 |
| | | **Hibernation history** | | | |
| | | Second-time | -0.035 | -3.61 | **<0.001** |
| | | Fixed effects removed: sex ($F_{1,63.5}$=1.22, $P$=0.273); treatment x sex ($F_{2,62.7}$=1.41, $P$=0.252); treatment x hibernation history ($F_{2,72.4}$=0.386, $P$=0.681) | | | |

| Response variable | Treatment comparison | Estimates | 95% CI | *P* |
|---|---|---|---|---|
| BCI emergence | Cold – Fluctuating | -0.012 | -0.029, 0.004 | 0.157 |
| | Mild – Cold | 0.003 | -0.013, 0.020 | 0.872 |
| | Mild – Fluctuating | -0.009 | -0.026, 0.008 | 0.377 |
| BCI recovery | Cold – Fluctuating | -0.010 | -0.033, 0.013 | 0.566 |
| | Mild – Cold | 0.012 | -0.012, 0.037 | 0.436 |
| | Mild – Fluctuating | 0.003 | -0.022, 0.027 | 0.965 |

Pairwise comparisons between treatments were performed using *emmeans* with Tukey's method for p-value adjustment. Body Condition Index (BCI) was calculated as the residuals from sex-specific linear regressions of $log_{10}$-transformed body mass on $log_{10}$-transformed snout–vent length (SVL)

**Table S5. Effect of treatments on changes in oxidative status indices in common wall lizards: LMs and post-hoc results.**

| **Response variable** | ***N*** | **Fixed effects** | **Estimates** | ***t*** | ***P*** |
|---|---|---|---|---|---|
| Total antioxidant capacity (mMol) | 23 | **(Intercept)** | 1.43 | 4.62 | **0.006** |
| | | **Treatment** | | | |
| | | Mild | 0.11 | 0.23 | 0.826 |
| | | Fluctuating | -0.24 | -0.50 | 0.632 |
| | | **BCI recovery** | 12.40 | 2.69 | **0.018** |
| | Fixed effects removed: hibernation history ($F_{1,17.1}$=0.007, $P$=0.933) | | | | |
| Malondialdehyde (µMol) | 37 | **(Intercept)** | 14.90 | 9.19 | **<0.001** |
| | | **Treatment** | | | |
| | | Mild | 2.27 | 0.90 | 0.381 |
| | | Fluctuating | 0.58 | 0.23 | 0.819 |
| | | **BCI recovery** | 3.27 | 1.34 | 0.191 |
| | Fixed effects removed: hibernation history ($F_{1,30.7}$=0.06, $P$=0.810) | | | | |
| Oxidised guanine species (pg ml$^{-1}$) | 36 | **Treatment** | | | 0.050 |
| | | Intercept | 381.41 | 7.52 | **<0.001** |
| | | **Mild** | 191.30 | 2.44 | **0.021** |
| | | Fluctuating | 65.74 | 0.86 | 0.396 |
| | Fixed effects removed: hibernation history ($F_{1,35}$=0.59, $P$=0.448); BCI recovery ($F_{1,35}$=0.60, $P$=0.444) | | | | |

| **Response variable** | **Treatment comparison** | **Estimates** | **95% CI** | ***P*** |
|---|---|---|---|---|
| Total antioxidant capacity (mMol) | Cold – Fluctuating | 0.25 | -1.29, 1.78 | 0.913 |
| | Mild – Cold | 0.11 | -1.41, 1.64 | 0.981 |
| | Mild – Fluctuating | 0.36 | -1.26, 1.98 | 0.842 |
| Malondialdehyde (µMol) | Cold – Fluctuating | -0.58 | -7.83, 6.68 | 0.977 |
| | Mild – Cold | 2.27 | -5.07, 9.61 | 0.713 |
| | Mild – Fluctuating | 1.70 | -5.87, 9.26 | 0.839 |
| Oxidised guanine species (pg ml$^{-1}$) | Cold – Fluctuating | -65.7 | -254, 122.10 | 0.669 |
| | Mild – Cold | 191.3 | -1.73, 384 | 0.053 |
| | Mild – Fluctuating | 125.6 | -78.14, 329 | 0.298 |

Pairwise comparisons between treatments were performed using *emmeans* with Tukey's method for p-value adjustment. Body Condition Index (BCI) was calculated as the residuals from sex-specific linear regressions of $\log_{10}$-transformed body mass on $\log_{10}$-transformed snout–vent length (SVL). Significant p-values (<0.05) are shown in bold.

Journal of Experimental Biology • Supplementary information
